# Diurnal differences in cerebral microvascular blood flow and oxygen delivery across brain regions in awake mice

**DOI:** 10.1117/1.NPh.13.1.015002

**Published:** 2026-01-06

**Authors:** Baoqiang Li, Hewei Cao, Qi Pian, Yimeng Wu, Jason E. Porter, Buyin Fu, Srinivasa Rao Allu, Sergei A. Vinogradov, Cenk Ayata, Ken Arai, Emiri T. Mandeville, Elga Esposito, Eng H. Lo, Sava Sakadžić

**Affiliations:** a Chinese Academy of Sciences, Brain Cognition and Brain Disease Institute, Shenzhen Institutes of Advanced Technology, Shenzhen, China; b Massachusetts General Hospital, Harvard Medical School, Athinoula A. Martinos Center for Biomedical Imaging, Charlestown, Massachusetts, United States; c University of Pennsylvania, Perelman School of Medicine, Department of Biochemistry and Biophysics, Philadelphia, Pennsylvania, United States; d University of Pennsylvania, School of Arts and Sciences, Department of Chemistry, Philadelphia, Pennsylvania, United States; e Massachusetts General Hospital, Harvard Medical School, Departments of Radiology and Neurology, Charlestown, Massachusetts, United States

**Keywords:** cerebral microvascular blood flow, partial pressure of oxygen, two-photon microscopy, optical coherent tomography, watershed, diurnal variation

## Abstract

**Significance:**

Elucidating the diurnal differences in cerebral hemodynamics is essential for both advancing our understanding of brain function and improving therapeutic strategies for neurological disorders. However, it remains unclear how the inactive and active diurnal phases influence the microvascular-scale distributions of cerebral blood flow and oxygenation and whether these distributions exhibit brain-region-specific variations.

**Aim:**

We aim to characterize the time-of-day variations in cerebral microvascular blood flow and oxygenation across brain regions in awake mice.

**Approach:**

We used two-photon microscopy and Doppler optical coherence tomography to quantify the resting-state microvascular blood flow and oxygenation parameters in the cerebral middle-cerebral-artery (MCA) territory and adjacent watershed area in the head-restrained, awake mice, during the inactive and active phases.

**Results:**

Microvascular blood flow was consistently higher during the inactive phase compared with the active phase. Specifically, this elevated flow reached statistical significance in the watershed area. Furthermore, oxygen extraction fraction increased in the MCA territory during the active phase but decreased in the watershed area.

**Conclusions:**

We reveal diurnal differences in cerebral microvascular blood flow and oxygenation, with the watershed area exhibiting a greater response to this effect. These findings underscore the potential of chronotherapeutic strategies to enhance treatment efficacy for cerebrovascular disorders.

## 1 Introduction

Many neurological disorders, including autism, depression, and Parkinson’s disease, are associated with the disruption of circadian rhythm.^1^ Taking individual chronotype into account is now crucial for improving drug efficacy and facilitating translational research.^2^ Notably, ischemic stroke occurrence peaks in the morning time (e.g., from 6:00 AM to 12:00 PM).^3^^,^^4^ Studies in rodent stroke models show that neuroprotective treatments are significantly more efficient when administered during the inactive diurnal phase (e.g., Zeitgeber time (ZT) = 3 to 9) rather than the active phase (e.g., ZT15-21).^5^ In addition, infarct size was smaller when stroke was induced during the active phase,^5^ suggesting that cerebral blood flow (CBF), which was reported to exhibit significant diurnal variations,^6^^,^^7^ may influence treatment outcomes. Interestingly, disruptions to the normal circadian rhythm can alter CBF patterns, increasing the risk of neurological and cardiovascular disorders.^8^^–^^14^ For instance, people who work night shifts or those with irregular sleep schedules face higher stroke risks due to impaired circadian-associated regulation of CBF and blood pressure.^15^^–^^17^

Therefore, elucidating diurnal variations in CBF is crucial not only for advancing our understanding of brain physiology but also for developing novel chronotherapeutic approaches for neurological disorders.^18^^–^^21^ However, gaps remain in our understanding of how the distributions of CBF and oxygenation vary by time of day at the microvascular level and whether these distributions differ across distinct brain regions. Of particular interest is the border-zone cortical tissue (a.k.a., watershed area), a region distal to the major arterial supply and typically situated beneath the cortical pial collaterals, where oxygen supply–demand mismatch and internal infarction have been observed.^22^^–^^24^ These findings suggest that the cerebral watershed area may exhibit distinct patterns of variations in cerebral microvascular blood flow and oxygenation. Therefore, there is an urgent need to investigate the diurnal differences in microvascular blood flow and oxygenation between the major-artery-supplied territory and the watershed area.

In this work, we employed two-photon microscopy (2 PM) to measure vascular partial pressure of oxygen (${\mathrm{PO}}_{2}$) and capillary red-blood-cell (RBC) flux, combined with Doppler optical coherence tomography (OCT) to quantify blood flow in the surfacing venules located near the cortical surface. These measurements were performed in the cerebral cortex in the middle-cerebral-artery (MCA) territory, as well as in the adjacent watershed area between the anterior cerebral artery (ACA) and MCA territories, in the head-restrained, awake C57BL/6 mice, during the inactive (e.g., ZT5-7) and active phases (e.g., ZT17-19). Due to the superior imaging penetration provided by the red-shifted fluorophore (i.e., Alexa680), measurements of capillary RBC flux were performed down to the subcortical white matter. Analysis of these data revealed that cerebral microvascular blood flow was consistently lower during the active phase compared with the inactive phase. Notably, this flow variation was significantly more pronounced in the watershed area than in the MCA territory. Intriguingly, we observed elevated oxygen extraction fraction (OEF) during the active phase relative to the inactive phase in the MCA territory, whereas the watershed area exhibited an opposite trend, combined with flow reduction, suggesting a reduced oxygen metabolism in the watershed area during the active phase. Collectively, these findings provide novel insights into diurnal variations in the regional distributions of cerebral microvascular blood flow and oxygen delivery under physiological conditions.

## 2 Materials and Methods

### 2.1 Animal Preparation

C57BL/6 mice were used in this work (n=6, female, 3 to 5 months old, body weight 20 to 25 g; Charles River Laboratories). To image brain tissue optically, a round-shaped glass window (3 mm in diameter) was implanted in the left hemisphere with the coordinates of AP = −1.5 mm relative to bregma and ML = −3.0 mm (i.e., centered approximately over the E1 whisker barrel). A metal head-post was glued to the skull above the right hemisphere, enabling head-restraint by connecting the head-post to a custom-designed cradle. Upon completion of the cranial surgery, mice were allowed 5 days to recover, followed by a 2-week training for habituating to the head-restraint condition in the cradle under the 2 PM microscope. During the habituation training, the duration of head-fixation was gradually increased from 10 min to 2 h. Although head-restrained, the mice were able to freely readjust their body position. Sweetened milk was provided as a reward at 20- to 25-min intervals during both training and subsequent imaging sessions. In line with our previously established procedures,^25^^,^^26^ mouse behavior was monitored using a webcam. The animals displayed natural grooming behavior and showed no overt signs of stress during imaging. Experiments were paused or terminated promptly if any indications of significant discomfort or anxiety were observed. The surgical and habituation-training procedures have been described in greater detail in our previous works.^25^^,^^26^ The above experimental procedures have been approved by the Massachusetts General Hospital Institutional Animal Care and Use Committee, in accordance with the Guide for the Care and Use of Laboratory Animals of the National Institutes of Health.

### 2.2 Experimental Timeline

The measurements of RBC flux, vascular ${\mathrm{PO}}_{2}$, and Doppler OCT were performed in the same animal cohort on separate days over a consecutive 3-day period. The imaging experiments were conducted during both the inactive and active phases of the mice. The mice were first housed with a regular light schedule (e.g., room light in the animal holding facility was ON from 7:00 AM to 7:00 PM, and OFF from 7:00 PM to 7:00 AM). The measurements were performed between noon and 2 PM (i.e., ZT6 ±1; inactive phase). Diurnal cycle was then modulated by reversing the light schedule in the holding room, such that the room light was ON from 7:00 PM to 7:00 AM, and OFF from 7:00 AM to 7:00 PM. The mice were given at least 3 weeks to adjust to the new light schedule, and then, the imaging experiments were conducted with the same group of mice between noon and 2 PM (i.e., ZT18 ±1; active phase). A more detailed description of the strategy for reversing the diurnal cycle can be found in our previous work.^5^

### 2.3 2-Photon Microscope

**Fig. 1:**
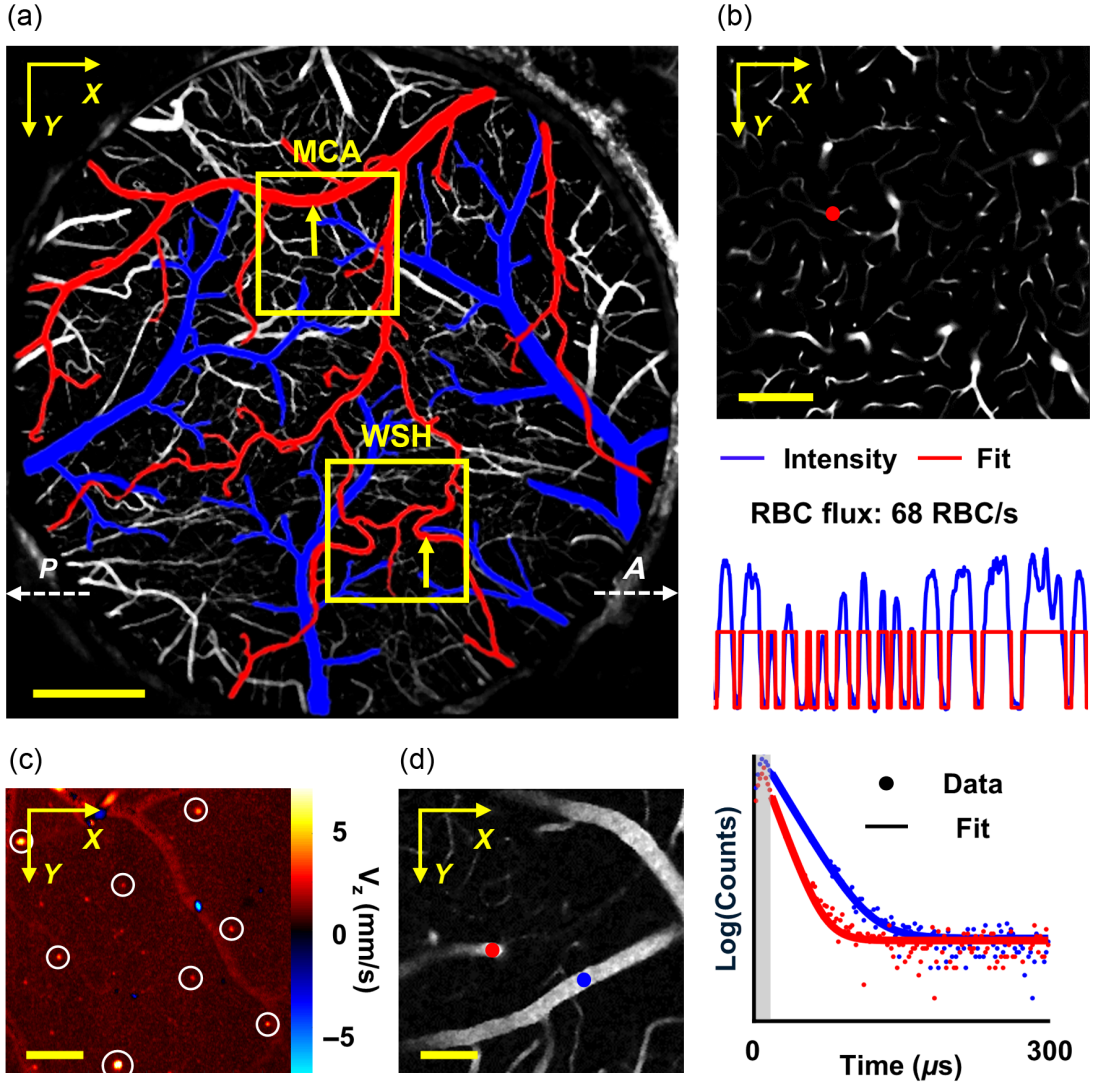
Multimodal imaging methods. (a) Maximum intensity projection (depth range of 0 to 100  μm) of a 2PM angiogram ($3.0\times 3.0\;mm^{2}$ FOV, Alexa680 labeling) acquired with a 4× objective in a representative mouse. Anatomical orientation is indicated by the dashed white arrows (A, anterior; P, posterior). Pial vasculature is pseudo-colored (red: arteries and pial collaterals; blue: veins). Yellow squares enclose the imaging ROIs in the MCA territory and watershed (WSH) area, with the yellow arrows indicating representative MCA and pial-collateral segments. Scale bar: 500  μm. (b) Upper panel: A representative two-dimensional angiogram acquired in the cortical gray matter (GM; depth: 0.6 mm). Lower panel: A representative 0.25-s-long fluorescence intensity time course acquired in a GM capillary, with the measurement location (red dot) indicated in the upper image. The flux is calculated as 68  RBC/s. Scale bar: 100  μm. (c) A two-dimensional Doppler OCT image of the cortical surface, with some representative venules (highlighted by white circles) selected for blood flow analysis. The color map encodes axial flow velocity, where positive values indicate blood flow out of the brain parenchyma (venous outflow), whereas negative values represent blood flow into the brain parenchyma (arterial inflow). Scale bar: 200  μm. (d) Left panel: A two-dimensional phosphorescence (Oxyphor2P) angiogram. Right panel: Two representative phosphorescence decay curves (logarithmic photon counts) from an arteriole (red; τ=12.53  μs, ${\mathrm{PO}}_{2}=93.1\;\mathrm{mmHg}$) and a venule (blue; τ=20.20  μs, ${\mathrm{PO}}_{2}=39.3\;\mathrm{mmHg}$), with the measurement locations marked by the red and blue dots, respectively, in the angiogram. The sampled photon counts (dots) and corresponding single-exponential fits (solid curves) are shown. Scale bar: 100  μm.

A home-built upright two-photon microscope was employed in this study.^27^^,^^28^ The system was powered by a commercial femtosecond laser (InSight DeepSee, Spectra-Physics; 680 to 1300 nm tuning range, ∼120  fs pulse width, 80 MHz repetition rate), with the output power modulated by an external electro-optic modulator (EOM) (ConOptics). The laser beam was scanned laterally (e.g., in the X and Y directions) using a pair of galvanometer mirrors (6215H, Cambridge Technology), expanded by a scan lens and tube lens combination, and finally focused by a water-immersion objective lens (XLUMPLFLN20XW, Olympus; numerical aperture 1.0, working distance 2.0 mm). For large-scale imaging, such as cortical surface microvasculature mapping [e.g., Fig. 1(a)], an air-spaced 4× objective lens (XLFLUOR4X/340, Olympus; numerical aperture 0.28, working distance 29.25 mm) was employed. For axial scanning, a linear motorized stage (M-112.1DG, Physik Instrumente) precisely positioned the objective lens along the Z-axis, enabling imaging at different focal planes. The emitted photons were first selectively reflected by a dichroic mirror (FF875-Di01-38.1×51.0, Semrock), then filtered through an infrared blocker (FF01-890/SP-25, Semrock) and an emission filter (FF01-709/167-25 or FF01-795/150-25, Semrock), before being detected by a photomultiplier tube (PMT) module (H10770PA-50, Hamamatsu). The PMT output signals were processed through a discriminator (C9744, Hamamatsu) before being acquired by a digital I/O board (NI PCle-6537, National Instruments) for subsequent analysis. To maintain physiological conditions, an electric heating system (TC-HLS-05, Bioscience Tools) was used to regulate objective lens temperature, ensuring the water between the cranial window and objective lens remained at 36°C to 37°C throughout all the imaging experiments.

### 2.4 2PM Imaging of Capillary RBC Flux

For RBC flux imaging, blood plasma was labeled using a near-infrared fluorophore Alexa680 conjugated to 70-kDa dextran. The dye exhibits a 2P excitation maximum at 1280 nm and a fluorescence emission peak at 700 nm. Approximately 20 min prior to imaging, we administered the Alexa680-dextran solution (0.1 mL of 5% w/v in PBS) via retro-orbital injection while mice were under brief anesthesia (1.5% to 2% isoflurane in an air/oxygen mixture, maintained for ∼2  min).

The RBC flux measurements were conducted at cortical depths of 400 and 600  μm within the gray matter. For the subcortical white matter, measurements were performed with 20 to 50  μm depth intervals within the white matter band, of which the upper and lower boundaries were delineated using an OCT-based approach.^29^ In each mouse, imaging regions of interest (ROIs) were selected in the cerebral MCA territory, as well as in the adjacent ACA-MCA watershed area, where the pial collaterals could be empirically identified [Fig. 1(a)]. At each imaging depth, a raster scan of fluorescence intensity was first conducted over a $0.5\times 0.5\;mm^{2}$ field of view (FOV) to visualize the microvascular network [Fig. 1(b)]. Capillaries were empirically identified as micro-vessels that did not exhibit penetrating trajectory (i.e., distinct from penetrating arterioles and venules) and had a vascular diameter of <10  μm, consistent with our previously established approach.^29^^,^^30^ The measurement locations were manually selected within the visually confirmed capillaries. During data acquisition, the excitation beam was parked at each measurement site for 0.5 s, allowing continuous excitation of Alexa680 fluorophore in the blood plasma. The resulting fluorescence signal was binned with 250-μs intervals, generating a 2000-point time series for subsequent analysis. Laser power and the duration of the “ON” phase during each 250-μs cycle were regulated by the EOM and maintained constant at each imaging plane. As the fluorophore was in the blood plasma but not in the RBCs, RBC passages through the excitation focal volume created fluctuations in fluorescence intensity. As illustrated in Fig. 1(b), “valleys” in the time course correspond to the RBC transits, whereas “peaks” represent plasma-filled intervals. The fluorescence time courses were then segmented using a binary thresholding algorithm,^29^ enabling direct calculation of RBC flux.

### 2.5 Doppler-OCT Imaging

A spectral-domain OCT system was used in this study.^31^^–^^33^ The system was equipped with a dual-superluminescent-diode light source (LS2000B, Thorlabs; center wavelength = 1310 nm, bandwidth = 170 nm). In biological tissue, the system provided an axial resolution of 3.5  μm, a pixel size of 2.8  μm, and an imaging depth of 1.6 mm along the Z-axis. A 10× objective lens (Plan Apo NIR, Mitutoyo) was used, yielding a lateral resolution of 3.5  μm in biological tissue.

Doppler OCT imaging was performed over a $1\times 1\;mm^{2}$ lateral FOV, matching the ROIs selected for the 2 PM measurements of RBC flux and ${\mathrm{PO}}_{2}$. We utilized scanning steps of 0.26 and 1.95  μm along the X (fast axis) and Y (slow axis) directions, respectively. Axial flow velocity ($V_{z}$) was derived from Doppler OCT data using a Kasai autocorrelation method.^34^ Blood flow of each vessel was calculated by integrating $V_{z}$ across the cross-sectional area, manually delineated to fully enclose the vascular lumen [Fig. 1(c)].^35^ To minimize artifacts from high-speed flow, analysis was restricted to superficial venules located within the top 100-μm depth under the cortical surface, excluding arterioles prone to aliasing effects.^35^ Each ROI was sampled with 20 repeated volumetric acquisitions. Volumetric OCT data were additionally used to estimate cortical and subcortical white matter thickness, enabling classification of RBC flux measurements into gray matter and white matter subgroups. The thickness quantification method followed our previously established works.^29^^,^^36^

### 2.6 2PM Imaging of Intravascular PO_2_

We used an oxygen-sensitive phosphorescence probe, Oxyphor2P. This probe exhibits a 2P excitation maximum at 950 nm and a phosphorescence emission peak at 757 nm.^37^ Approximately 20 min before imaging experiments, each mouse received a retro-orbital injection of 0.1 mL of Oxyphor2P solution (∼34  μM in PBS) under brief anesthesia (1.5% to 2% isoflurane in an air/oxygen mixture, duration of ∼2  min).

The intravascular ${\mathrm{PO}}_{2}$ measurements were conducted from the cortical surface to a depth of 500  μm with 100  μm depth intervals. In each mouse, imaging ROIs were selected within both the MCA territory and the adjacent ACA-MCA watershed area, corresponding spatially with the ROIs chosen for RBC flux measurements. At each imaging plane, we initially performed a raster scan of phosphorescence intensity within a $0.5\times 0.5\;mm^{2}$ FOV to visualize the microvascular structure, followed by manual selection of measurement sites in the visually identifiable microvascular segments [Fig. 1(d)]. At each measurement location, Oxyphor2P molecules were excited with a 10-μs-long laser excitation gated by EOM, followed by a 290-μs-long detection of phosphorescence emission. Such a 300-μs-long excitation/decay cycle was repeated 2000 times (corresponding to an acquisition duration of 0.6 s) to obtain a high signal-to-noise-ratio phosphorescence decay profile [Fig. 1(d)]. Phosphorescence lifetime was determined by a nonlinear least square fitting of a single-exponential decay model.^27^ Lifetime was ultimately converted to absolute ${\mathrm{PO}}_{2}$ using an established Stern–Volmer calibration.^37^

### 2.7 Calculations of SO_2_ and OEF

The hemoglobin oxygen saturation (${\mathrm{SO}}_{2}$) was calculated using the Hill equation with parameters specific for C57BL/6 mice (Hill coefficient h=2.59, $P_{50}=40.2\;\mathrm{mmHg}$).^38^ The $P_{50}$ represents the ${\mathrm{PO}}_{2}$ at which hemoglobin is 50% saturated. The OEF was determined as $({\mathrm{SO}}_{2,A}\text{-}{\mathrm{SO}}_{2,V})/{\mathrm{SO}}_{2,A}$, where ${\mathrm{SO}}_{2,A}$ and ${\mathrm{SO}}_{2,V}$ denote arterial and venous ${\mathrm{SO}}_{2}$, respectively. For OEF calculations in this study, we utilized ${\mathrm{PO}}_{2}$ measurements obtained from cortical arterioles and venules located within the superficial 100-μm depth.

### 2.8 Data Analysis

The experimental design and reporting of this study comply with the ARRIVE guidelines. All data are presented as mean ± STD, where applicable. Statistical analyses of both absolute measurements and relative changes were performed using paired Student’s t-test (MATLAB, MathWorks Inc.), with statistical significance (P<0.05) indicated by the asterisk symbol (*).

**Fig. 2:**
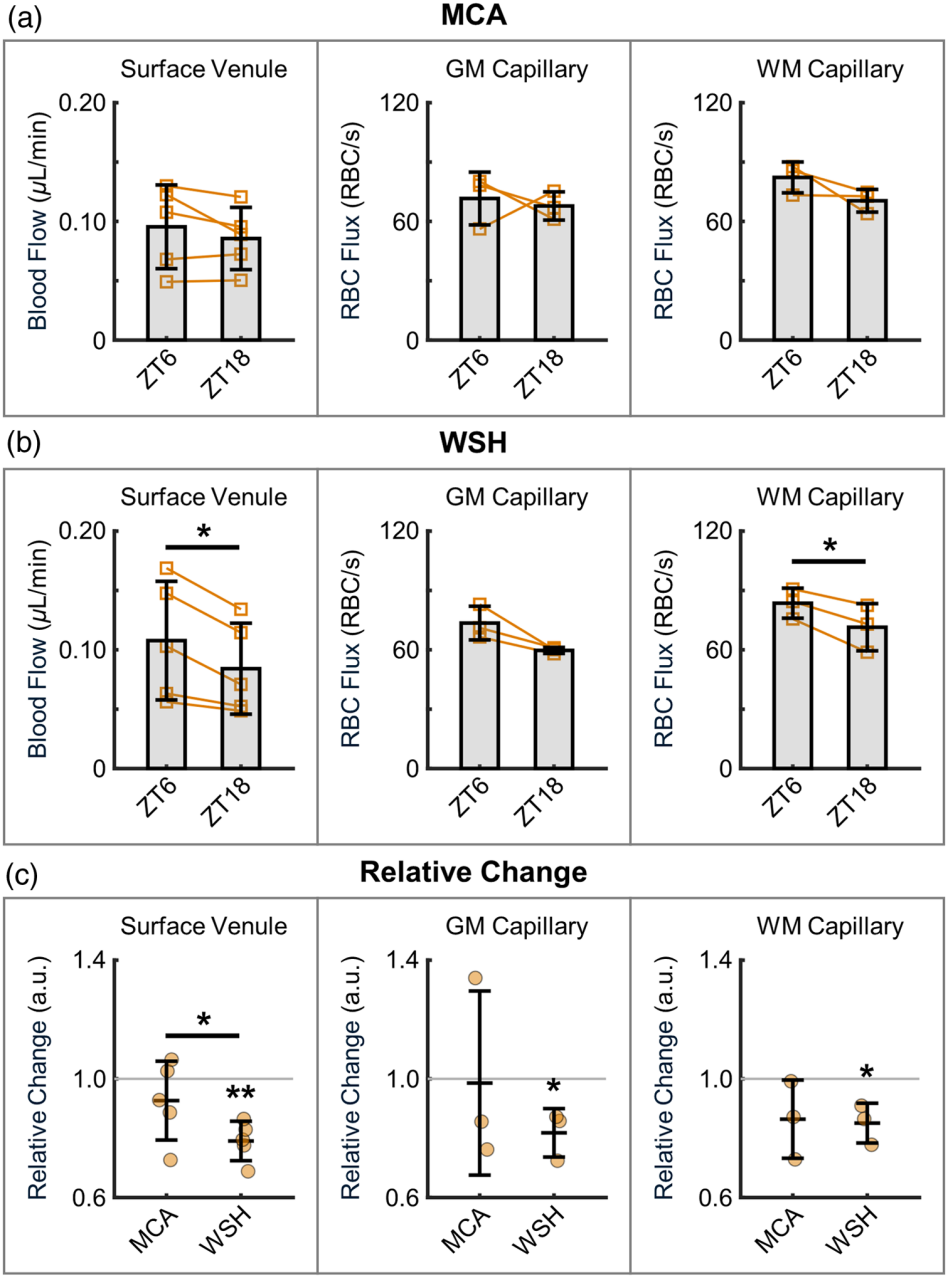
Cerebral microvascular blood flow. (a) Measurements in the MCA territory: venular blood flow (left), capillary RBC flux in the gray matter (GM; middle), and in the subcortical white matter (WM; right). (b) Corresponding measurements in the watershed area (WSH). Left panels in (a) and (b): Venular blood flow was measured within the top 100  μm below the cortical surface in 44 and 65 surfacing venules in the MCA territory and WSH area, respectively, across n=5 mice. Measurements from the same vessels at both diurnal phases were selected for analysis. Orange squares represent the mean flow values of individual mice, with lines connecting the mean values within the same mice between the two phases. The group means were first calculated per mouse and then averaged across all mice. Middle and right panels in (a) and (b): Capillary RBC flux was measured in GM at depths of 400 and 600  μm, as well as in WM at an average depth of 800  μm, across n=3 mice. In the MCA territory, 226 and 237 GM capillaries were selected at ZT6 and ZT18, respectively; and 218 and 167 WM capillaries were selected at ZT6 and ZT18, respectively. In the WSH area, 329 and 457 GM capillaries were selected at ZT6 and ZT18, respectively; and 194 and 218 WM capillaries were selected at ZT6 and ZT18, respectively. The measurements were performed in the same depths/ROIs, which might lead to some degree of overlap of the sampled capillaries. Performing measurements in the same capillaries is challenging because small changes in head tilt between experiments can lead to substantial difficulties in identifying and selecting the same capillaries. Orange dots represent the RBC flux values from individual capillaries across all mice. (c) Relative changes in venular blood flow, GM RBC flux, and WM RBC flux, calculated from the same data in panels (a) and (b). Orange circles represent the mean values of individual mice. The group means were first calculated per mouse and then averaged across all mice. Data are expressed as mean ± STD. Asterisks denote statistical significance (*P<0.05, **P<0.01).

**Fig. 3:**
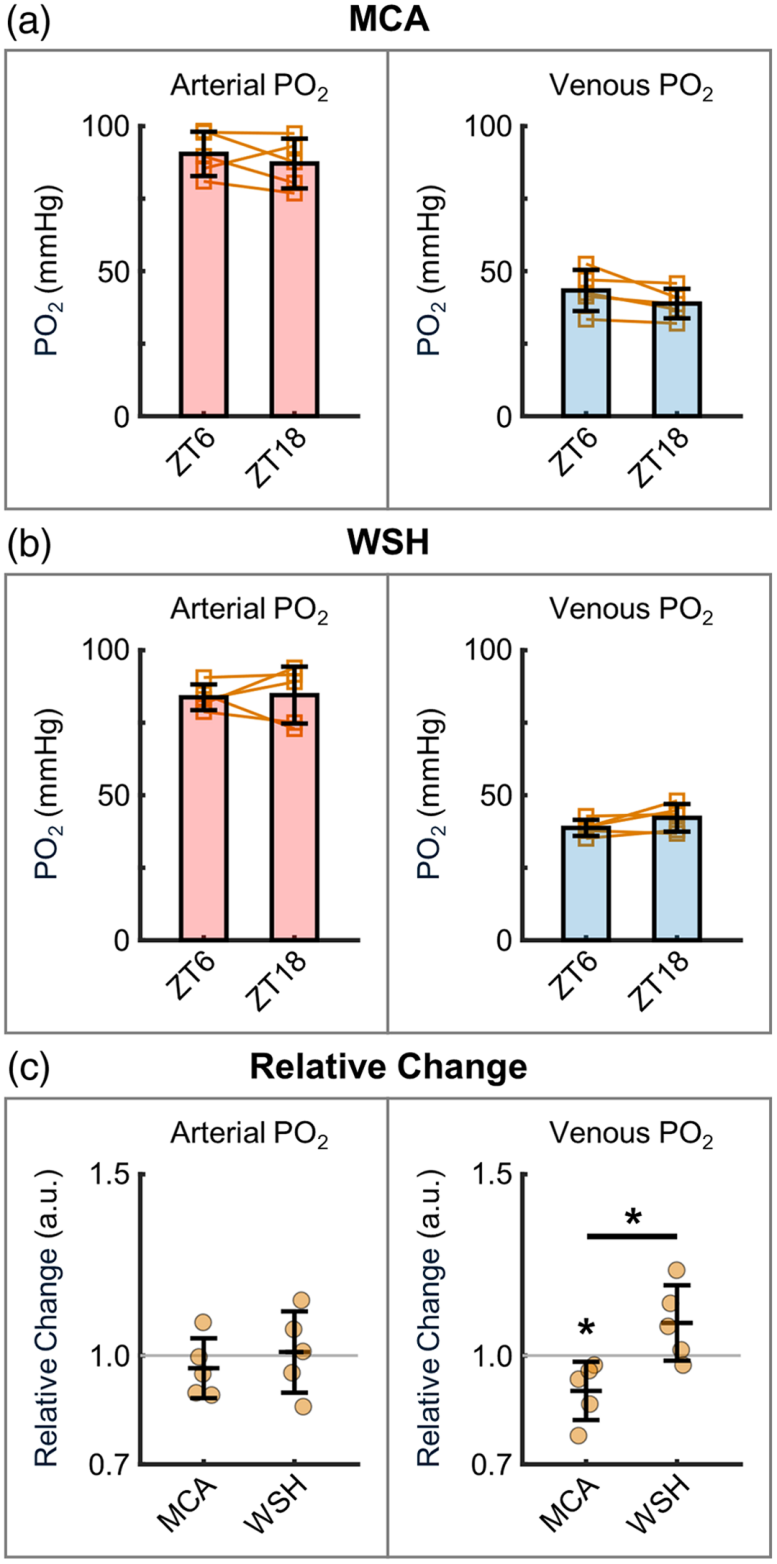
Cerebral vascular ${\mathrm{PO}}_{2}$. (a) Measurements in the MCA territory: ${\mathrm{PO}}_{2}$ in the diving arterioles (left) and surfacing venules (right) located within the top 100-μm depth under the cortical surface. (b) Corresponding measurements in WSH. Left panels in (a) and (b): the measurements were performed in 32 and 41 arterioles in the MCA territory and WSH area, respectively, across n=5 mice. Right panels in (a) and (b): the measurements were performed in 47 and 37 venules in the MCA territory and WSH area, respectively, in the same mice. Measurements from the same vessels at both diurnal phases were selected for analysis. Orange squares represent the mean ${\mathrm{PO}}_{2}$ values of individual mice, with lines connecting the mean values within the same mice between the two phases. The group means were first calculated per mouse and then averaged across all mice. (c) Relative changes in arterial and venous ${\mathrm{PO}}_{2}$, calculated from the same data in panels (a) and (b). Orange circles represent the mean values of individual mice. The group means were first calculated per mouse and then averaged across all mice. Data are expressed as mean ± STD. The asterisk symbol denotes statistical significance (*P<0.05).

**Fig. 4:**
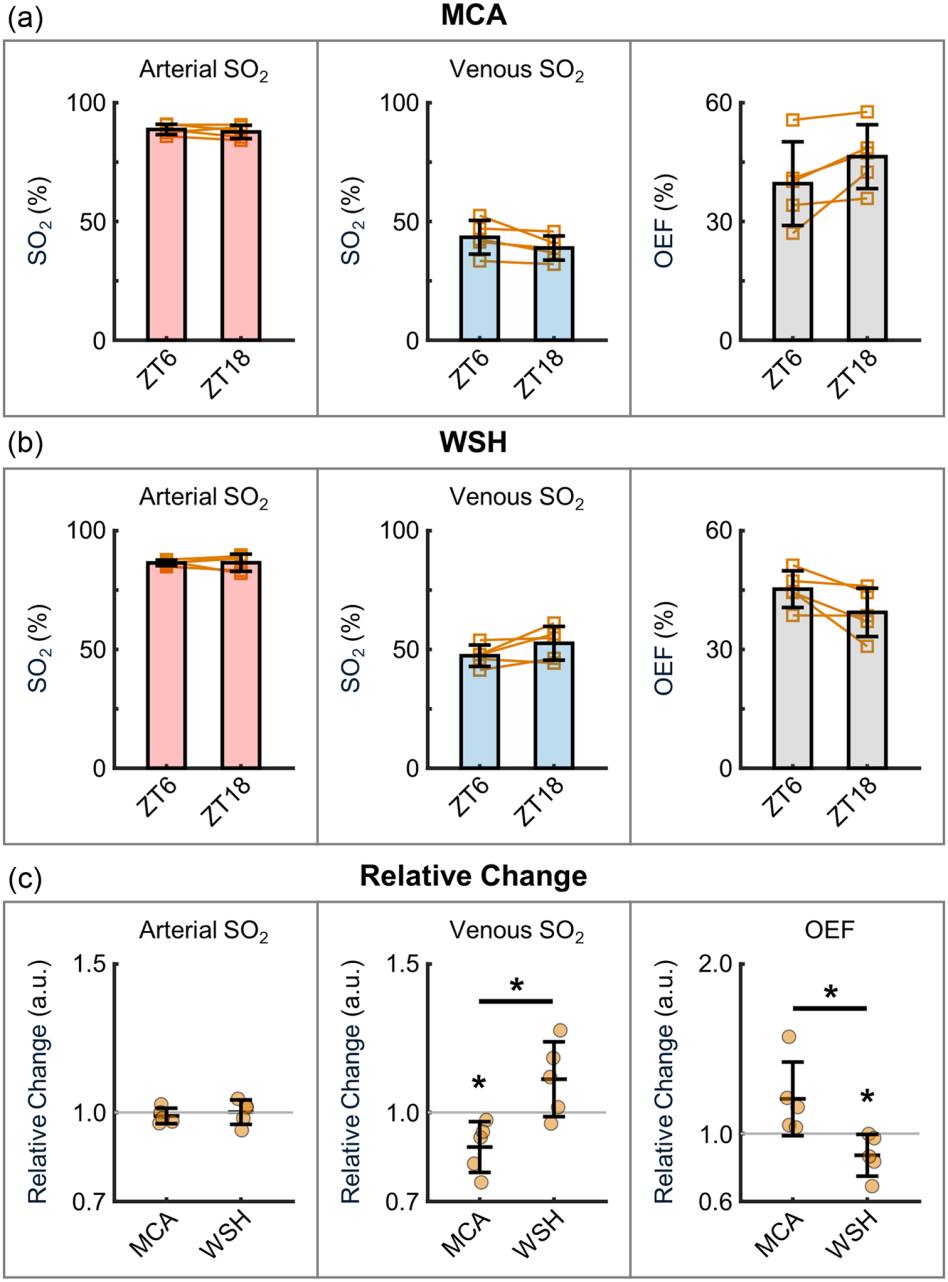
Cerebral vascular ${\mathrm{SO}}_{2}$ and OEF. (a)–(b) Arterial/venous ${\mathrm{SO}}_{2}$ (left and middle) and OEF (right) calculated from the ${\mathrm{PO}}_{2}$ data in Fig. 3. Orange squares indicate the mean values for individual mice, with lines connecting the mean values from the same mice across the two phases. The group means were first calculated per mouse and then averaged across all mice. (c) Relative changes in arterial/venous ${\mathrm{SO}}_{2}$ and OEF, calculated from the same dataset shown in panels (a) and (b). Orange circles represent the mean values for individual mice. The group means were first calculated per mouse and then averaged across all mice. Data are expressed as mean ± STD. The asterisk symbol denotes statistical significance (*P<0.05).

**Fig. 5:**
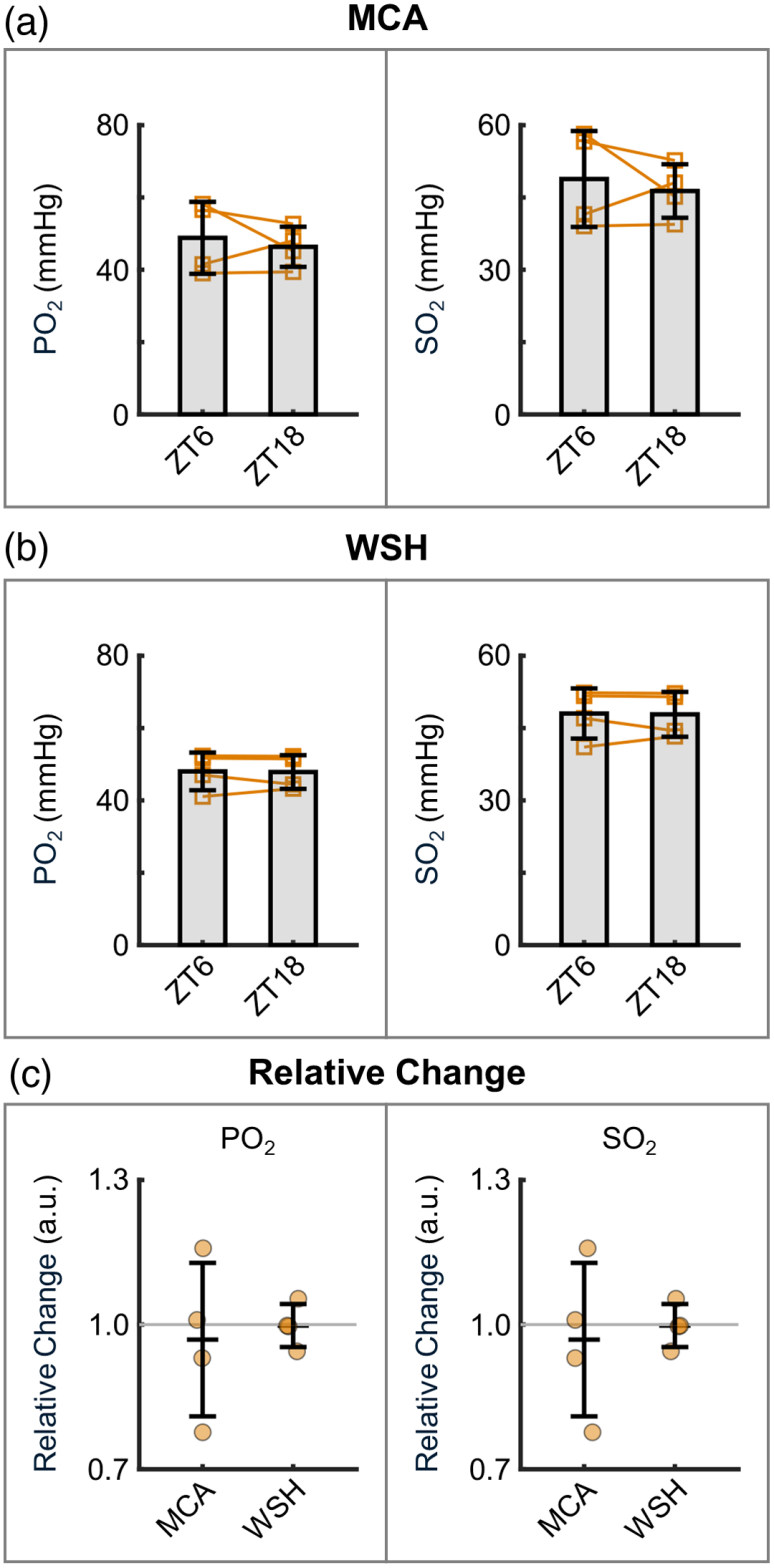
Cerebral capillary oxygenation. (a)–(b) Capillary ${\mathrm{PO}}_{2}$ (left) and ${\mathrm{SO}}_{2}$ (right) in the MCA (a) and WSH (b) regions. The measurements were performed at depths ranging from 100 to 500  μm below the cortical surface with 100-μm intervals, across n=4 mice. At ZT6, 567 and 398 capillaries were sampled in the MCA territory and WSH area, respectively; at ZT18, 856 and 491 capillaries were sampled in the MCA territory and WSH area, respectively. The measurements were performed in the same depths/ROIs, which might lead to some degree of overlap of the sampled capillaries. Performing measurements in the same capillaries is challenging because small changes in head tilt between experiments can lead to substantial difficulties in identifying and selecting the same capillaries. Orange dots represent pooled ${\mathrm{PO}}_{2}$ values from individual capillaries across all mice. (c) Relative changes in capillary ${\mathrm{PO}}_{2}$ and ${\mathrm{SO}}_{2}$, calculated from the same dataset in panels (a) and (b). Orange circles indicate the mean values for individual mice. The group means were first calculated per mouse and then averaged across all mice. Data are expressed as mean ± STD. No statistical significance was found.

Leveraging measurements obtained from the same vessels (e.g., venular blood flow with Doppler OCT, and arterial/venous ${\mathrm{PO}}_{2}$ in the cortical surface) and/or from consistent ROIs and imaging depths (e.g., capillary RBC flux and capillary ${\mathrm{PO}}_{2}$) across diurnal cycles, we analyzed the relative changes of each parameter between ZT6 and ZT18. Specifically, the relative change was calculated as the ratio of values measured at ZT18 to those at ZT6, using the same absolute datasets as specified in the respective figure legends. The calculation procedures are as below:

(1) For venular blood flow [left panel, Fig. 2(c)], cortical-surface vascular ${\mathrm{PO}}_{2}$ [Fig. 3(c)] and ${\mathrm{SO}}_{2}$ [first two left panels, Fig. 4(c)]: Ratios were first computed for individual vessels between diurnal cycles in each mouse, then averaged per mouse, and finally across all mice.
(2) For capillary RBC flux [middle and right panels, Fig. 2(c)], as well as capillary ${\mathrm{PO}}_{2}$ and ${\mathrm{SO}}_{2}$ [Fig. 5(c)]: Mean absolute values were first calculated per mouse. The ratio between diurnal cycles was then computed for each mouse and subsequently averaged across all mice.
(3) For OEF [rightmost panel, Fig. 4(c)]: ${\mathrm{SO}}_{2}$ of each vessel (arteriole or venule) was derived from its corresponding ${\mathrm{PO}}_{2}$ value. Mean arteriolar and venous ${\mathrm{SO}}_{2}$ were calculated per mouse from all selected vessels, after which OEF was computed for each mouse. The relative change between diurnal cycles was determined per mouse and then averaged across all mice.

The relative change values greater than one indicate increases in the measured parameters from ZT6 to ZT18, whereas values less than one indicate decreases. For within-group comparisons, Student’s t-test was used to assess whether the mean ratios significantly differed from unity, with statistical significance denoted by the asterisk symbol (*) above the corresponding bars. Between-group comparisons were also carried out using Student’s t-test.

## 3 Results

### 3.1 Diurnal Differences in Microvascular Blood Flow Are Significantly Larger in the Watershed Area Than in the MCA Territory

Using Doppler OCT and 2 PM, we quantified microvascular blood flow parameters in both the MCA territory and the adjacent ACA-MCA watershed (WSH) area during the inactive (ZT6) and active (ZT18) diurnal phases.

In the MCA territory, venular blood flow showed a modest though nonsignificant decrease from 0.095±0.035  μL/min at ZT6 to 0.086±0.026  μL/min at ZT18 [Fig. 2(a), left]. A similar trend was observed in capillary RBC flux. Specifically, RBC flux in the gray matter (GM) capillaries decreased slightly from 71.57±13.34  RBC/s at ZT6 to 67.80±7.13  RBC/s at ZT18 [Fig. 2(a), middle]. By contrast, RBC flux in the white matter (WM) capillaries exhibited a more pronounced decline, i.e., from 82.23±7.80  RBC/s at ZT6 to 70.43±5.75  RBC/s at ZT18 [Fig. 2(a), right].

In the watershed area, venular blood flow decreased significantly (P=0.016) from 0.108±0.05  μL/min at ZT6 to 0.084±0.038  μL/min at ZT18 [Fig. 2(b), left]. A notable reduction was also observed in GM RBC flux, which declined from 73.53±8.51  RBC/s at ZT6 to 59.70±1.54  RBC/s at ZT18 [P=0.094; Fig. 2(b), middle]. Strikingly, WM RBC flux decreased significantly (P=0.040) from 83.57±7.58  RBC/s at ZT6 to 71.43±11.93  RBC/s at ZT18 [Fig. 2(b), right].

Further analysis revealed a significant relative reduction in venular blood flow in the watershed area. The extent of this relative change differed significantly between the two imaging regions [Fig. 2(c), left]. In addition, the relative changes in both GM and WM RBC flux in the watershed area also reached statistical significance [Fig. 2(c), middle and right].

### 3.2 Diurnal Differences in Cerebral Vascular Oxygenation Exhibit Distinct Patterns Between the MCA Territory and Watershed Area

We next employed 2 PM combined with Oxyphor2P to evaluate the diurnal variations in cerebral vascular ${\mathrm{PO}}_{2}$. In the MCA territory, arterial ${\mathrm{PO}}_{2}$ showed a modest decrease from 90.4±7.6  mmHg at ZT6 to 87.1±8.6  mmHg at ZT18 [Fig. 3(a), left]. Conversely, venous ${\mathrm{PO}}_{2}$ notably declined from 43.3±7.1  mmHg at ZT6 to 38.8±5.1  mmHg at ZT18 [P=0.08; Fig. 3(a), right]. In the WSH area, arterial ${\mathrm{PO}}_{2}$ exhibited a slight increase from 83.8±4.4mmHg at ZT6 to 84.5±9.8  mmHg at ZT18 [Fig. 3(b), left]. By contrast, venous ${\mathrm{PO}}_{2}$ rose noticeably from 38.7±2.8  mmHg at ZT6 to 42.2±4.8  mmHg at ZT18 [Fig. 3(b), right].

The changes of ${\mathrm{SO}}_{2}$ [Figs. 4(a)–4(b), left and middle], derived from the same ${\mathrm{PO}}_{2}$ datasets in Fig. 3, displayed consistent trends. We further evaluated the OEF calculated from ${\mathrm{SO}}_{2}$. In the MCA territory, OEF showed an obvious increase from 39.6±10.6% at ZT6 to 46.4±8.1% at ZT18, although this difference did not reach statistical significance [P=0.053; Fig. 4(a), right]. By contrast, an opposite trend was observed in the WSH area, where OEF decreased from 45.2±4.6% at ZT6 to 39.4±6.1% at ZT18 [P=0.076; Fig. 4(b), right]. Analysis of relative changes [Fig. 4(c)] revealed a statistically significant difference in relative OEF changes between the MCA and WSH regions. This divergence in OEF patterns likely stems from the opposing venular oxygenation trends in these two regions.

Finally, we assessed capillary oxygenation across multiple cortical depths (e.g., 100 to 500  μm with 100-μm intervals). Both regions exhibited subtle variations [Figs. 5(a)–5(b)]. Specifically, in the MCA territory, capillary ${\mathrm{PO}}_{2}$ declined from 48.84±9.96  mmHg at ZT6 to 46.34±5.55  mmHg at ZT18, whereas capillaries in the WSH area showed a comparable reduction from 48.03±5.20  mmHg at ZT6 to 47.85±4.65  mmHg at ZT18. Capillary ${\mathrm{SO}}_{2}$ exhibited similar patterns. Analysis of relative changes indicated only minor variations between the two phases [Fig. 5(c)].

## 4 Discussion and Conclusion

We conducted comprehensive steady-state measurements using 2 PM and Doppler OCT to systematically evaluate the diurnal changes in cerebral microvascular blood flow and microvascular oxygenation in both the MCA territory and watershed area under physiological conditions. The ability to detect these subtle yet significant changes with a minimized number of animals was enabled by our precise and quantitative imaging techniques, coupled with rigorously designed experimental strategies for consistently imaging the same vessels or in the same ROIs and imaging depths across imaging sessions. To minimize complexity in interpreting the results arising from anatomical variability in the posterior cerebral artery in mice, we specifically selected the watershed area between the ACA and MCA territories. All the parameters were measured in awake mice, thereby avoiding the confounding effects of anesthesia on neuronal activity, brain hemodynamics, and metabolism.^39^^–^^42^ Furthermore, conducting imaging experiments in the awake state helps eliminate the influences of sleep on cerebral hemodynamics.^43^^,^^44^

We initially evaluated the changes in venular blood flow using Doppler OCT. During the inactive phase (ZT6), the measured venular blood flow was 0.095±0.035  μL/min in the MCA territory and 0.108±0.05  μL/min in the watershed area (Fig. 2). This result does not necessarily mean that CBF in the watershed area is higher than in the MCA territory as CBF measurements in individual surfacing venules are highly dependent on the caliber of the vessels selected for analysis. The measured flow values align reasonably well with the previously reported range of 0.03-0.1  μL/min measured in awake mice.^36^^,^^45^^,^^46^ Minor discrepancies may arise from variations in mouse age and genetic background. Notably, venular flow decreased during the active phase in both regions, reaching 0.086±0.026  μL/min in the MCA territory and 0.084±0.038  μL/min in the watershed area, with the reduction being statistically significant in the latter. The observed higher flow during the inactive phase is consistent with a previous study reporting vascular dilation at ZT6 compared with ZT18.^47^ Given that changes in white matter capillary RBC flux in the watershed area followed a similar trend, it is plausible that the observed reduction in venous flow may be partly mediated by the deeply penetrating principal cortical venules that drain the deepest cortical regions and white matter.^48^

Next, we measured capillary RBC flux in the deeper layers at 2 PM. During the inactive phase, RBC flux in the gray matter (68.2±10.7  RBC/s in the MCA territory and 68.0±6.3  RBC/s in the watershed area; Fig. 2) is comparable with the previously reported values obtained in awake mice, using similar 2 PM-based approaches.^25^^,^^36^^,^^45^^,^^49^ The measurements performed during the inactive phase showed that capillary RBC flux was higher in the white matter than in the gray matter at both imaging locations (75.7±8.7  RBC/s in the MCA territory and 77.1±9.3  RBC/s in the watershed area), consistent with our prior observations in both anesthetized and awake mice.^29^^,^^36^ This difference likely stems from the lower vascular resistance in the white matter microvasculature, which results in higher RBC flux per capillary segment despite lower overall blood perfusion but significantly lower capillary density.^50^^–^^52^ During the active phase, capillary RBC flux was decreased in both the gray and white matter within the MCA territory. This finding further supports our earlier work demonstrating that cerebral white matter is more susceptible to physiological perturbations.^29^ The heightened vulnerability of white matter to hypoperfusion largely arises from its distal position in the arterial tree.^29^^,^^53^^,^^54^ This effect might be even more pronounced in the watershed area—located at the terminal ends of arterial supply networks—where the RBC flux changes in both gray and white matter were substantially greater than those in the MCA territory.

Furthermore, we assessed the vascular oxygenation changes at 2 PM. During the inactive phase in the MCA territory, the measured arterial and venular ${\mathrm{PO}}_{2}$ values were 90.4±7.6  mmHg and 43.3±7.1  mmHg at the cortical surface (Fig. 3), whereas capillary ${\mathrm{PO}}_{2}$ in the deeper cortical layers was 45.0±9.9  mmHg (Fig. 5). These results are consistent with prior measurements in age-matched C57BL/6 mice.^25^^,^^49^ Interestingly, we observed a distinct brain-region-specific OEF pattern (Fig. 4). In the MCA territory, OEF increased from the inactive phase to the active phase, potentially compensating for the reduced blood flow to maintain stable oxygen metabolism. Conversely, in the watershed area, OEF decreased alongside reduced flow, suggesting compromised oxygen consumption. Taken together, we observed diurnal-phase-associated fluctuations in microvascular blood flow, vascular oxygenation, and OEF in both the MCA and WSH regions, with the WSH area exhibiting more extensive changes in the measured parameters. Although the magnitude of these diurnal variations is modest, they may still hold physiological significance—particularly in metabolically vulnerable regions such as the watershed white matter, which possesses limited metabolic reserve. Moreover, if such diurnal fluctuations are chronically attenuated or lost, the resulting disruption of circadian regulation could adversely affect brain function and potentially contribute to disease onset or unfavorable clinical outcomes. These considerations highlight an important open question in cerebrovascular physiology and warrant further investigation in future studies.

This study has several limitations that should be acknowledged. First, our Doppler OCT measurements were restricted to venules due to potential aliasing artifacts in arterioles caused by their higher flow speed. However, we posit that venular measurements alone provide sufficient data for intergroup comparisons as blood flow should be approximately conserved between the arteriolar and venular networks within the examined brain regions.^55^ This conservation principle supports the validity of our venule-focused approach for detecting relative flow changes across diurnal phases. Employing an OCT system with a higher line rate or applying the phase unwrapping algorithms could enable flow measurements in arterioles. Second, capillary identification was based on morphological criteria (diameter ≤10  μm) for RBC flux and capillary ${\mathrm{PO}}_{2}$ measurements. We implemented stringent selection criteria ($R^{2}\geq 0.5$) to ensure the analyzed vessels exhibited single-file flow patterns, thereby effectively excluding small arterioles and venules from our capillary analysis. Third, despite determining cortical thickness in the imaged ROIs using an OCT-based approach,^29^ we cannot exclude the possibility that some of the RBC-flux measurements, particularly those acquired near the cortical-white matter boundary, may have included capillaries draining the deepest parts of the layer VIb in the cortical gray matter.

**Fig. 6:**
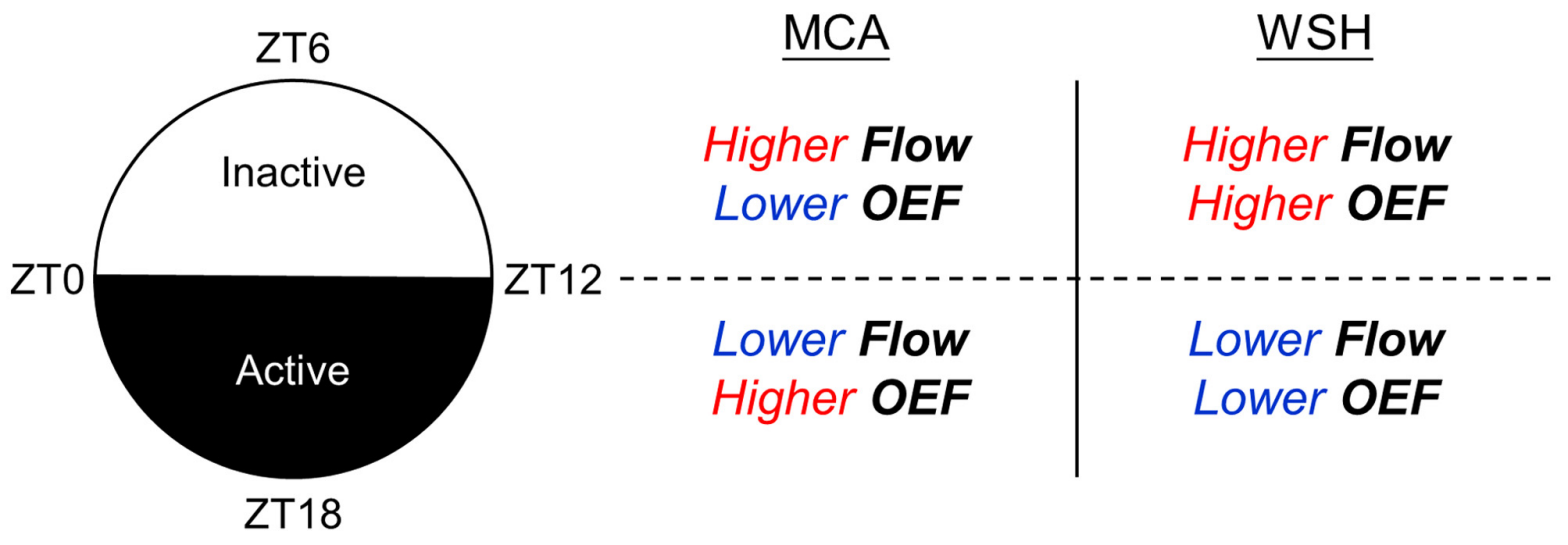
Schematic summary of key findings. In mice, cerebral microvascular blood flow and OEF display distinct change patterns between the inactive (ZT6) and active (ZT18) phases across the two brain regions.

In summary, our study reveals the time-of-day-dependent redistributions of cerebral microvascular blood flow and oxygenation delivery (Fig. 6). We demonstrate that microvascular blood flow—assessed by venous flow and capillary RBC flux—is consistently higher during the inactive phase in both the MCA territory and the adjacent ACA-MCA watershed area. By contrast, a regional divergence in OEF was observed: it was decreased in the MCA territory but conversely increased in the watershed area during the inactive phase. A combination of increased blood flow and OEF implies increased oxygen metabolism in the watershed area during the inactive phase. Collectively, these findings suggest that cerebral watershed white matter may be inherently vulnerable to physiological perturbations. This vulnerability may explain the frequent occurrence of internal watershed infarction in the elderly, particularly under conditions of prolonged hypoperfusion. Furthermore, the fact that this increased oxygen metabolism and potential vulnerability appear to be maximal during the inactive phase may be consistent with emerging observations that brain injury after cerebral ischemia is more severe during the daytime in rodent stroke models (the inactive phase for nocturnal rodents) and more severe during the nighttime for clinical stroke patients (the inactive phase for diurnal humans).^5^^,^^11^^,^^13^^,^^56^

## Data Availability

The data supporting the findings of this study are available from the authors upon reasonable request.
